# Age-stratified colonoscopy outcomes and referral-factor associations in the Swedish colorectal cancer fast-track pathway: a retrospective cohort study

**DOI:** 10.1177/17562848261460138

**Published:** 2026-06-28

**Authors:** Abdullah Jajan, Michiel van Nieuwenhoven

**Affiliations:** Division of Gastroenterology, Department of Internal Medicine, Faculty of Medicine and Health, Örebro University, Örebro, Sweden; Division of Gastroenterology, Department of Internal Medicine, Faculty of Medicine and Health, Örebro University, Södra Grev Rosengatan, Örebro SE 70182, Sweden; University Health Care Research Unit, Faculty of Medicine and Health, Örebro University, Örebro, Sweden

**Keywords:** age groups, colonoscopy findings, colorectal cancer, positive predictive value, standardised course of care

## Abstract

**Background:**

The standardised course of care colorectal cancer pathway (SCC-CRC) is a symptom-based fast-track diagnostic pathway. Age-related differences in outcomes and referral-factor associations within different age groups are incompletely described.

**Objective:**

To describe colonoscopy outcomes across age groups in SCC-CRC and evaluate unadjusted and adjusted associations between referral factors and CRC and advanced colorectal lesions.

**Design:**

A retrospective cohort study.

**Methods:**

A cohort of 3258 patients referred through SCC-CRC and investigated with colonoscopy during 2016–2023 was grouped by age (18–40, 41–50, 51–60, 61–70, 71–80, >80 years). Referral factors were analysed using positive predictive value (PPVs), sensitivity, specificity, likelihood ratios and logistic regression adjusted for age, sex and coexisting referral factors; a second model included faecal immunochemical test (FIT) among patients with available FIT data.™

**Results:**

CRC was detected in 0% of patients aged 18–40, 6.5% aged 41–50, 11.2% aged 51–60, 18.3% aged 61–70, 18.1% aged 71–80 and 21.2% aged >80. The frequency of referral factors varied across age groups, while the PPV for CRC increased with age. Rectal and radiological findings were associated with CRC across age groups. In adjusted analysis without FIT, radiological findings, rectal findings, anaemia and visible blood were independently associated with CRC. Among patients with FIT data, positive FIT was strongly associated with CRC and negative FIT had a high negative predictive value.

**Conclusion:**

CRC detection increased with age in this symptomatic cohort. Several referral factors were independently associated with CRC, but PPV increased with age, partly because CRC prevalence increased with age. Routine FIT testing in SCC-CRC referrals and complete FIT data capture should be assessed in future studies.

## Plain language summary

How often colonoscopy finds colorectal cancer at different ages in patients with warning symptoms in Sweden

Colorectal cancer is more common in older people, but it can also occur in younger adults. In Sweden, patients with symptoms that may suggest colorectal cancer can be referred quickly for investigation through a fast-track pathway. In this study, we looked at how often colonoscopy found cancer and other colorectal pathology in patients of different ages who were referred through this pathway. We studied 3,258 patients who underwent colonoscopy between 2016 and 2023. We divided patients into age groups and examined which symptoms and test results were linked to cancer or other findings. We found that no cancers were detected in patients under 40 years of age. However, cancer was found in 6.5% of patients aged 41–50 and 11.4% of those aged 51–60. The chance of finding cancer increased steadily with age. We also found that the types of symptoms leading to referral differed between age groups. For example, younger patients more often had visible blood in the stool, while older patients more often had changes in bowel habits. Some referral factors, such as findings during rectal examination, results from imaging, and stool tests for blood (FIT), were linked to a higher chance of cancer. However, other symptoms were also linked to non-cancer conditions, meaning they are not specific for cancer. These results show that bowel cancer can occur in symptomatic patients below the current screening age. They also suggest that improving the use of stool tests (FIT) in symptomatic patients may help to better prioritize who should undergo colonoscopy.

## Introduction

Colorectal cancer (CRC) is the third most common cancer in Sweden and the third leading cause of cancer-related mortality worldwide. Although CRC risk increases with age, with a median diagnostic age of 73 years for colon cancer and 70 years for rectal cancer in Sweden,^1,2^ incidence among younger adults has risen in several countries.^3^ The causes of this trend remain unclear, though lifestyle-related factors such as dietary patterns and physical inactivity have been suggested.^4^

To reduce mortality and improve prognosis, many countries have implemented fast-track diagnostic pathways for CRC.^5^ In Sweden, the standardised course of care for CRC (SCC-CRC) was introduced in 2016 and is based on five referral criteria: visible blood in stool not explained by recto-proctoscopy or persistent bleeding despite treatment, unexplained iron-deficiency anaemia, altered bowel habits for more than 4 weeks in patients above 40 years combined with a positive faecal immunochemical test (FIT), rectal findings suggestive of CRC and radiological findings suggestive of CRC.^6^ SCC-CRC is a symptom-based diagnostic pathway for patients with clinical suspicion of CRC and differs from population-based screening, which is solely based on FIT testing. The SCC-CRC criteria have undergone revisions over time, especially regarding altered bowel habits, and previous studies have shown that the predictive value of individual criteria varies.^5^ In parallel with SCC-CRC, Sweden’s national CRC screening programme has been gradually introduced since 2021, with nationwide implementation by 2026 for individuals aged 60–74 years, using biennial testing.^7^

For patients with suspected CRC, national guidelines recommend colonoscopy within 10 days, which remains the diagnostic gold standard.^6^ Approximately 120,000 colonoscopies are performed annually in Sweden, a proportion of which occur within the SCC-CRC pathway.^8^ However, most examinations do not reveal significant pathology, contributing to a substantial burden on the healthcare system.^9^ Moreover, several studies have reported that SCC-CRC has not substantially improved CRC detection rates or prognosis since its introduction. In Region Örebro County (RÖC), 37.5% of CRC cases were diagnosed through the routine waiting list rather than SCC-CRC, showing limitations in current referral criteria.^10,11^

While referral through SCC-CRC is permitted from 40 years of age, population-based screening begins at 60 years. Age-specific colonoscopy outcomes and the independent value of SCC-CRC referral factors are incompletely described. It remains unclear whether the same referral factors have similar associations with CRC across age groups, or whether observed positive predictive value (PPVs) mainly reflect the age-related rise in CRC prevalence. Hence, we examined referral factors and endoscopic outcomes within the SCC-CRC cohort across different age groups and described how CRC prevalence develops with increasing age.

## Methods

### Study design and patient material

This was a retrospective cohort study, and all data were collected from a database in RÖC. The database contained endoscopy data from all three hospitals in RÖC from 2016 to 2023 and included referral information (SCC-CRC criteria), available laboratory values, age, sex and colonoscopy findings. Patients were selected consecutively from accepted SCC-CRC referrals to the endoscopy unit. No random sampling was used. Reporting follows the Strengthening the Reporting of Observational Studies in Epidemiology (STROBE) statement.^12^ During the COVID-19 pandemic, only SCC referrals were accepted; therefore, SCC-CRC referral rates were comparable to the pre-pandemic period.^5^ All colonoscopies were performed by experienced gastroenterologists and colorectal surgeons.

If symptoms were not mentioned in the referral, they were assumed to be absent. All colonoscopy findings were recorded. Pathological findings included: CRC in right colon or transverse colon, CRC in left colon or rectum, synchronous CRC, total CRC, high-grade dysplasia (HGD) polyps, low-grade dysplasia polyps, high-risk adenomas (HRA), diverticulosis, unspecified inflammation, suspected inflammatory bowel disease, microscopic colitis, diverticulitis, haemorrhoids and angiodysplasia. Absence of pathological findings was also recorded in the database. HRA was defined as ⩾3 adenomas, adenomas ⩾10 mm, HGD, villous histology or serrated polyps with either ⩾10 mm in size or dysplasia. FIT was analysed separately; if one or more FITs were positive, then it was recorded as positive. Other alarm symptoms included unintentional weight loss, B-symptoms and rectal bleeding without prior rectoscopy. Radiological findings were coded as a binary referral factor because the source database did not contain details on the imaging method, finding type or level of suspicion.

Most SCC-CRC referrals came from general practitioners. SCC-CRC can also be initiated following a routine colonoscopy with suspected cancer. This study, however, only included SCC-CRC referrals to the endoscopy unit. All SCC-CRC referrals were reviewed by gastroenterologists at the University Hospital Örebro before acceptance.

Patients were stratified into six age groups: 18–40, 41–50, 51–60, 61–70, 71–80 and >80 years.

The primary outcomes of the study were the prevalence of CRC across age groups and unadjusted and adjusted associations between signs and symptoms and cancer. The secondary outcomes comprise the prevalence of other pathological findings and differences in signs and symptoms, as well as the PPVs and odds ratios (ORs) for these findings across age groups.

### Ethical considerations

This study was approved by the Swedish Ethical Review Authority (Dnr. 2023-03776-02) and was conducted in accordance with the Declaration of Helsinki and applicable Swedish regulations. The Regional Ethics Committee granted a waiver of informed consent due to the retrospective design, minimal risk to participants and use of de-identified data prior to analysis. Data processing complied with the General Data Protection Regulation and the Swedish Patient Data Act; only authorised personnel had access to identifiable records.

### Statistical analysis

IBM SPSS Statistics (version 31.0.0.0) was used for all analyses. PPVs and ORs with 95% confidence intervals (CI) were calculated. PPV was defined as the proportion of colonoscopies with a given referral factor that had a pathological finding. ORs were calculated as the odds of a pathological finding among patients with the referral factor divided by the odds among patients without the referral factor. Mean age was calculated and reported as mean ± standard deviation (SD). Sensitivity, specificity, negative predictive value (NPV), positive and negative likelihood ratios (LR+ and LR−) were calculated for CRC. PPV was interpreted together with CRC prevalence, because PPV depends on disease prevalence.

The changes in SCC-CRC entry criteria were not adjusted for during analysis, since a previous study showed that these changes did not significantly affect referral rates or increase CRC incidence. However, the most recent alteration of adding FIT may have influenced referral patterns, as studies have shown that a positive FIT has a high PPV for CRC and a very high NPV (approximately 98%).^5,13,14^

Categorical variables were analysed using 2 × 2 crosstabulations. Pearson’s Chi-square test was used or Fisher’s exact test if any parameter had an expected frequency <5. A *p*-value <0.05 was considered statistically significant. ORs with 95% CIs were calculated from the same tables. In comparisons where a 2 × 2 contingency table contained a zero cell, ORs could not be estimated reliably and were therefore not reported. In two comparisons, the Chi-square test yielded *p* < 0.05 even though the CI included 1, indicating statistical uncertainty. Fisher’s exact test *p*-values were reported for these sparse tables (marked with ‘*’).

Multivariable logistic regression was performed for CRC. The base model included age, sex, visible blood, anaemia, altered bowel habits, rectal findings, radiological findings and other alarm symptoms. A second complete-case model added FIT status among patients with available FIT data. Results are reported as adjusted ORs (aORs) with 95% CIs. Patients with and without FIT data were compared using a *t*-test for age and Chi-square tests for categorical variables.

Missing data were handled by complete-case analysis for each specific analysis. Referral factors were coded as present when documented in the referral information and absent when not documented. FIT analyses were restricted to patients with available FIT results. Patients without FIT results were excluded from FIT-specific diagnostic accuracy calculations and from the complete-case FIT regression model. No imputation was performed.

No formal sample size calculation was performed because all eligible SCC-CRC colonoscopies in the regional database were included. The available sample size was considered adequate for the main CRC model because the number of CRC events exceeded conventional minimum event-per-variable recommendations for logistic regression.

## Results

**Figure 1.**
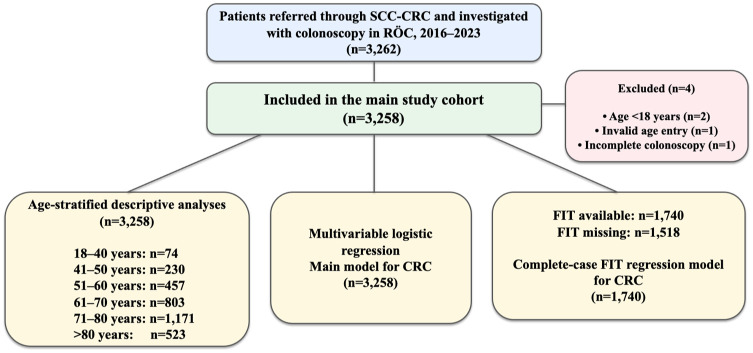
Flowchart of the study.

The database included SCC-CRC criteria, laboratory results and colonoscopy findings for 3262 patients from 3 hospitals in RÖC. Four patients were excluded: two patients were younger than 18 years, one record had an invalid age entry and one colonoscopy was incomplete. Hence, a total of 3258 patients were included. The flowchart is presented in Figure 1.

FIT results were available in 1740 of 3258 patients (53.4%) and were missing in 1518 patients (46.6%). Patients with and without FIT data differed in several clinical features (see Table S1). CRC was detected in 243 of 1740 patients with FIT data (14.0%) and in 293 of 1518 patients without FIT data (19.3%). Radiological findings and rectal findings were more frequent among patients without FIT data, whereas anaemia and altered bowel habits were more frequent among patients with FIT data. These differences indicate that FIT testing was selective rather than randomly missing.

As shown in Table 1, 83.7% of patients had a pathological colonoscopy finding, of which 19.7% had CRC, 4.7% had HGD polyps and 21.0% had HRA. Overall, 52.3% of the patients were female. Compared to females, males had a slightly higher proportion of pathological findings overall. SCC-CRC criteria associated with pathological findings showed high PPVs, but these PPVs should be interpreted in relation to the high prevalence of pathological colonoscopy findings in this selected cohort. The mean age was 70.3 years for patients with pathological findings and 61.0 years for patients without pathological findings.

**Table 1.** Frequencies, PPVs, ORs and *p*-values for patient characteristics and SCC-CRC referral factors in relation to pathological colonoscopy findings, stratified by age group.

All age groups	Pathological colonoscopy		PPV (%)	OR (95% CI)	*p-*Value
	Yes (*n* = 2726), *n* (%)	No (*n* = 532), *n* (%)			
Males	1323 (48.5)	230 (43.2)	85.2	1.24 (1.03–1.49)	0.025*
Females	1403 (51.5)	302 (56.8)	82.3	Reference	Reference
Mean age (years), *n* ±SD	70.3 ±11.5	61.0 ±14.2			
SCC criteria					
Visible blood	566 (20.8)	106 (19.9)	84.2	1.05 (0.84–1.33)	0.662
Anaemia	680 (24.9)	149 (28.0)	82.0	0.85 (0.69–1.05)	0.138
Altered bowel habits	1369 (50.2)	264 (49.6)	83.8	1.02 (0.85–1.23)	0.801
Rectal findings	414 (15.2)	39 (7.3)	91.4	2.26 (1.61–3.19)	<0.001*
Radiological findings	448 (16.4)	52 (9.8)	89.6	1.82 (1.34–2.46)	<0.001*
Other alarm symptoms	885 (32.5)	175 (32.9)	83.5	0.98 (0.80–1.20)	0.847
Positive FIT	1135 (78.7)	197 (66.1)	85.2	1.90 (1.45-2.49)	<0.001
18–40 years	Pathological colonoscopy		PPV (%)	OR (95% CI)	*p-*Value
	Yes (*n* = 40), *n* (%)	No (*n* = 34), *n* (%)			
Males	23 (57.5)	14 (41.2)	62.2	1.93 (0.76–4.88)	0.162
Females	17 (42.5)	20 (58.8)	45.9	Reference	Reference
SCC criteria					
Visible blood	17 (42.5)	10 (29.4)	63.0	1.77 (0.67–4.67)	0.244
Anaemia	8 (20.0)	10 (29.4)	44.4	0.60 (0.21–1.75)	0.347
Altered bowel habits	11 (27.5)	8 (23.5)	57.9	1.23 (0.43–3.53)	0.697
Rectal findings	8 (20.0)	2 (5.9)	80.0	4.00 (0.79–20.32)	0.097
Radiological findings	0 (0.0)	5 (14.7)			0.017*
Other alarm symptoms	19 (47.5)	13 (38.2)	59.4	1.46 (0.58–3.70)	0.423
Positive FIT	8 (66.7)	9 (64.3)	47.1	1.11 (0.22–5.63)	1.000
41–50 years	Pathological colonoscopy		PPV (%)	OR (95% CI)	*p-*Value
	Yes (*n* = 132), *n* (%)	No (*n* = 98), *n* (%)			
Males	73 (55.3)	41 (41.8)	64.0	1.72 (1.01–2.92)	0.043*
Females	59 (44.7)	57 (58.2)	50.9	Reference	Reference
41–50 years	Pathological colonoscopy		PPV (%)	OR (95% CI)	*p-*Value
	Yes (*n* = 132), *n* (%)	No (*n* = 98), *n* (%)			
SCC criteria					
Visible blood	41 (31.1)	25 (25.5)	62.1	1.32 (0.73–2.36)	0.357
Anaemia	17 (12.9)	22 (22.4)	43.6	0.51 (0.25–1.02)	0.056
Altered bowel habits	62 (47.0)	43 (43.9)	59.0	1.13 (0.67–1.92)	0.642
Rectal findings	23 (17.4)	11 (11.2)	67.6	1.67 (0.77–3.61)	0.190
Radiological findings	24 (18.2)	8 (8.2)	75.0	2.50 (1.07–5.84)	0.030*
Other alarm symptoms	47 (35.6)	27 (27.6)	63.5	1.45 (0.82–2.57)	0.196
Positive FIT	51 (78.5)	37 (69.8)	58.0	1.58 (0.68–3.62)	0.283
51–60 years	Pathological colonoscopy		PPV (%)	OR (95% CI)	*p*-Value
	Yes (*n* = 336), *n* (%)	No (*n* = 121), *n* (%)			
Males	158 (47.0)	48 (39.7)	76.7	1.35 (0.88–2.06)	0.163
Females	178 (53.0)	73 (60.3)	70.9	Reference	Reference
SCC criteria					
Visible blood	90 (26.8)	26 (21.5)	77.6	1.34 (0.81–2.20)	0.251
Anaemia	48 (14.3)	26 (21.5)	64.9	0.61 (0.36–1.04)	0.065
Altered bowel habits	175 (52.1)	62 (51.2)	73.8	1.03 (0.68–1.57)	0.873
Rectal findings	47 (14.0)	5 (4.1)	90.4	3.77 (1.46–9.72)	0.003*
Radiological findings	58 (17.3)	9 (7.4)	86.6	2.60 (1.24–5.42)	0.009*
Other alarm symptoms	105 (31.2)	49 (40.5)	68.2	0.67 (0.43–1.03)	0.065
Positive FIT	126 (75.4)	42 (61.8)	75.0	1.90 (1.04–3.48)	0.035*
61–70 years	Pathological colonoscopy		PPV (%)	OR (95% CI)	*p-*Value
	Yes (*n* = 690), *n* (%)	No (*n* = 114), *n* (%)			
Males	337 (48.8)	45 (39.5)	88.2	1.46 (0.98–2.19)	0.064
Females	353 (51.2)	69 (60.5)	83.6	Reference	Reference
SCC criteria					
Visible blood	147 (21.3)	19 (16.7)	88.6	1.35 (0.80–2.29)	0.257
Anaemia	145 (21.0)	32 (28.1)	81.9	0.68 (0.44–1.07)	0.092
Altered bowel habits	350 (50.7)	61 (53.5)	85.2	0.89 (0.60–1.33)	0.582
Rectal findings	104 (15.1)	13 (11.2)	88.9	1.38 (0.75–2.55)	0.303
Radiological findings	118 (17.1)	15 (13.2)	88.7	1.36 (0.76–2.43)	0.294
Other alarm symptoms	203 (29.4)	40 (35.1)	83.5	0.77 (0.51–1.17)	0.222
Positive FIT	278 (77.7)	37 (64.9)	88.3	1.88 (1.03–3.42)	0.037*
71–80 years	Pathological colonoscopy		PPV (%)	OR (95% CI)	*p*-Value
	Yes (*n* = 1045), *n* (%)	No (*n* = 125), *n* (%)			
Males	511 (48.9)	63 (50.4)	89.0	0.94 (0.65–1.36)	0.751
Females	534 (51.1)	62 (49.6)	89.6	Reference	Reference
SCC criteria					
Visible blood	199 (19.0)	21 (16.8)	90.5	1.16 (0.71–1.91)	0.544
Anaemia	291 (27.8)	45 (36.0)	86.6	0.69 (0.46–1.01)	0.057
Altered bowel habits	539 (51.6)	70 (56.0)	88.5	0.84 (0.58–1.22)	0.350
Rectal findings	158 (15.1)	5 (4.0)	96.9	4.28 (1.72–10.63)	<0.001*
Radiological findings	159 (15.2)	8 (6.4)	95.2	2.62 (1.26–5.48)	0.008*
Other alarm symptoms	337 (32.2)	32 (25.6)	91.3	1.38 (0.91–2.11)	0.131
Positive FIT	458 (78.4)	47 (59.5)	90.7	2.47 (1.52–4.04)	<0.001*
>80 years	Pathological colonoscopy		PPV (%)	OR (95% CI)	*p-*Value
	Yes (*n* = 483), *n* (%)	No (*n* = 40), *n* (%)			
Males	221 (45.8)	19 (47.5)	92.1	0.93 (0.49–1.78)	0.832
Females	262 (54.2)	21 (52.5)	92.6	Reference	Reference
SCC criteria					
Visible blood	72 (14.9)	5 (12.5)	93.5	1.23 (0.46–3.23)	0.680
Anaemia	171 (35.4)	14 (35.0)	92.4	1.02 (0.52–2.00)	0.959
Altered bowel habits	232 (48.0)	20 (50.0)	92.1	0.92 (0.48–1.76)	0.811
Rectal findings	74 (15.3)	3 (7.5)	96.1	2.23 (0.67–7.43)	0.180
Radiological findings	89 (18.4)	7 (17.5)	92.7	1.06 (0.46–2.49)	0.884
Other alarm symptoms	174 (36.0)	14 (35.0)	92.6	1.05 (0.53–2.06)	0.897
Positive FIT	214 (83.6)	25 (92.6)	89.5	0.41 (0.09–1.79)	0.276

Pathological colonoscopies include all pathologies mentioned under ‘Methods’. Visible blood refers to visible blood in stool. Rectal findings refer to suspected CRC at rectal examination (rectal palpation or recto-proctoscopy). Other alarm symptoms include unintentional weight loss, B-symptoms and rectal bleeding without rectoscopy.

CI, confidence interval; FIT, faecal immunochemical test; OR, odds ratio; PPV, positive predictive value; SCC-CRC, standardised course of care colorectal cancer pathway; SD, standard deviation; * = statistical significant difference.

**Figure 2.**
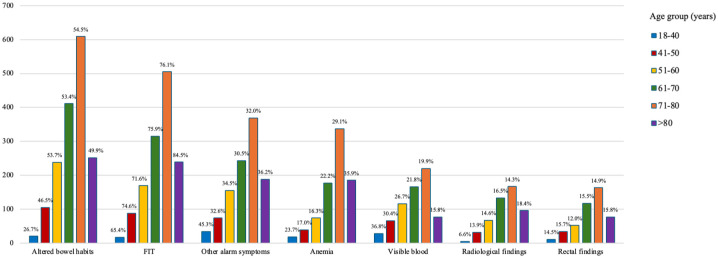
Frequency of referral factors and laboratory findings across age groups. The *y*-axis shows the absolute number of reported referral factors and laboratory findings, while percentages show prevalence within each age group. The distribution of referral factors differs across age groups. Visible blood refers to visible blood in stool. Rectal findings refer to suspected CRC at rectal examination (rectal palpation or recto-proctoscopy). CRC, colorectal cancer; FIT, faecal immunochemical test.

Figure 2 shows that visible blood in stool and other alarm symptoms were the most common symptoms in patients aged 18–40 years, whereas altered bowel habits were predominant in older age groups. Overall, the distribution of referral factors differed across age groups. Visible blood in stool was the least frequent symptom in patients older than 80 years, together with rectal findings. FIT was performed in 53.4% of patients, 76.6% of whom tested positive. Patients with available FIT differed from those without FIT in age, CRC prevalence and several referral factors, indicating possible selection in FIT testing.

**Figure 3.**
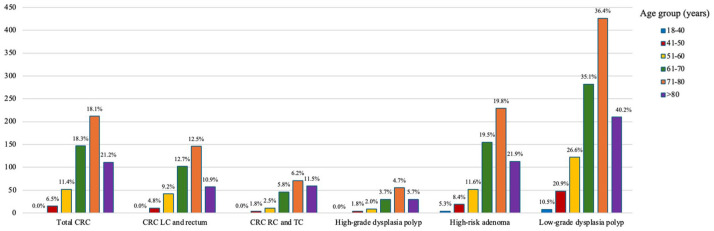
Frequency of CRC and colorectal lesions across age groups. The *y*-axis shows the absolute number of CRC cases and colorectal lesions, while percentages show prevalence within each age group. Synchronous CRC was excluded due to low numbers. CRC, colorectal cancer; LC, left colon; RC, right colon; TC, transverse colon.

**Figure 4.**
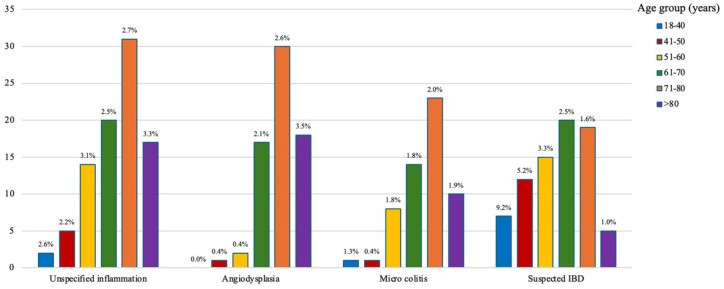
Frequency of other colonoscopy findings across age groups. The *y*-axis shows the absolute number of reported findings, while percentages show prevalence within each age group.

No CRC was detected in patients aged 18–40 years; therefore, sensitivity and LRs could not be estimated in this age group. CRC detection increased to 6.5% in those aged 41–50 years and reached 21.2% in patients older than 80 years, as seen in Figure 3. Rectal and left-sided colon cancers were more common than right-sided colon cancers in all age groups above 40 years, except in patients older than 80 years, in whom right- and transverse colon cancers predominated. The lowest prevalence of pathological findings was observed in patients aged 18–40 years. Haemorrhoids were the most common finding in patients aged 18–60 years, whereas diverticula predominated in patients aged 61 years and older, as depicted in Figure 4.

As shown in Table 2, the PPV for several referral factors increased with age, but this should be interpreted in relation to the higher CRC prevalence in older patients. Positive FIT was analysed only among patients with available FIT results. In diagnostic accuracy analyses for CRC, rectal findings and radiological findings had high specificity (88.3% and 87.2%) but low sensitivity (25.0% and 28.2%). Positive FIT, among patients with available FIT data, had a sensitivity 96.7%, specificity 26.7%, PPV 17.6%, NPV 98.0%, LR+ 1.32 and LR− 0.12. Results for HGD polyps and HRA are presented in Tables S2 and S3.

**Table 2.** Diagnostic accuracy measures for CRC by age group.

Age group	Referral factor	Factor present, *n*/*N*	CRC with factor/all CRC	PPV (%)	Sensitivity (%)	Specificity (%)	NPV (%)	LR+	LR−	OR (95% CI)	*p-*Value
18–40 y	Visible blood	27/74	0/0								
	Anaemia	18/74	0/0								
	Altered bowel habits	19/74	0/0								
	Rectal findings	10/74	0/0								
	Radiological findings	5/74	0/0								
	Other alarm symptoms	32/74	0/0								
	Positive FIT	17/26	0/0								
41–50 y	Visible blood	66/230	6/15	9.1	40.0	72.1	94.5	1.43	0.83	1.72 (0.59–5.05)	0.377
	Anaemia	39/230	3/15	7.7	20.0	83.3	93.7	1.19	0.96	1.24 (0.33–4.63)	0.724
	Altered bowel habits	105/230	6/15	5.7	40.0	54.0	92.8	0.87	1.11	0.78 (0.27–2.27)	0.649
	Rectal findings	34/230	4/15	11.8	26.7	86.0	94.4	1.91	0.85	2.24 (0.67–7.50)	0.248
	Radiological findings	32/230	6/15	18.8	40.0	87.9	95.5	3.31	0.68	4.85 (1.59–14.73)	0.009*
	Other alarm symptoms	74/230	5/15	6.8	33.3	67.9	93.6	1.04	0.98	1.06 (0.35–3.21)	1.000
	Positive FIT	88/118	7/7	8.0	100.0	27.0	100.0	1.37	0.00	–	0.189
51–60 y	Visible blood	116/457	12/51	10.3	23.5	74.4	88.6	0.92	1.03	0.89 (0.45–1.77)	0.747
	Anaemia	74/457	9/51	12.2	17.6	84.0	89.0	1.10	0.98	1.12 (0.52–2.42)	0.765
	Altered bowel habits	237/457	24/51	10.1	47.1	47.5	87.7	0.90	1.11	0.81 (0.45–1.44)	0.467
	Rectal findings	52/457	14/51	26.9	27.5	90.6	90.9	2.93	0.80	3.66 (1.82–7.38)	<0.001*
	Radiological findings	67/457	17/51	25.4	33.3	87.7	91.3	2.71	0.76	3.56 (1.85–6.84)	<0.001*
	Other alarm symptoms	154/457	20/51	13.0	39.2	67.0	89.8	1.19	0.91	1.31 (0.72–2.38)	0.376
	Positive FIT	168/235	20/21	11.9	95.2	30.8	98.5	1.38	0.15	8.92 (1.17–67.86)	0.012*
61–70 y	Visible blood	166/804	33/147	19.9	22.4	79.8	82.1	1.11	0.97	1.14 (0.74–1.76)	0.550
	Anaemia	177/804	40/147	22.6	27.2	79.1	82.9	1.30	0.92	1.42 (0.94–2.14)	0.093
	Altered bowel habits	411/804	69/147	16.8	46.9	47.9	80.2	0.90	1.11	0.81 (0.57–1.17)	0.262
	Rectal findings	117/804	41/147	35.0	27.9	88.4	84.6	2.41	0.82	2.96 (1.92–4.56)	<0.001*
	Radiological findings	133/804	41/147	30.8	27.9	86.0	84.2	1.99	0.84	2.38 (1.56–3.62)	<0.001*
	Other alarm symptoms	243/804	49/147	20.2	33.3	70.5	82.5	1.13	0.95	1.19 (0.81–1.75)	0.364
	Positive FIT	315/415	75/76	23.8	98.7	29.2	99.0	1.39	0.05	30.94 (4.24–225.62)	<0.001*
71–80 y	Visible blood	220/1170	49/212	22.3	23.1	82.2	82.8	1.29	0.94	1.38 (0.97–1.98)	0.076
	Anaemia	336/1170	67/212	19.9	31.6	71.9	82.6	1.13	0.95	1.18 (0.86–1.63)	0.305
	Altered bowel habits	609/1170	108/212	17.7	50.9	47.7	81.5	0.97	1.03	0.95 (0.70–1.28)	0.721
	Rectal findings	163/1170	53/212	32.5	25.0	88.5	84.2	2.18	0.85	2.57 (1.78–3.72)	<0.001*
	Radiological findings	167/1170	49/212	29.3	23.1	87.7	83.7	1.88	0.88	2.14 (1.47–3.11)	<0.001*
	Other alarm symptoms	369/1170	68/212	18.4	32.1	68.6	82.0	1.02	0.99	1.03 (0.75–1.42)	0.852
	Positive FIT	505/663	88/91	17.4	96.7	27.1	98.1	1.33	0.12	10.90 (3.40–34.97)	<0.001*
>80 y	Visible blood	77/523	11/111	14.3	9.9	84.0	77.6	0.62	1.07	0.58 (0.29–1.13)	0.107
	Anaemia	185/523	49/111	26.5	44.1	67.0	81.7	1.34	0.83	1.60 (1.05–2.46)	0.029*
	Altered bowel habits	252/523	44/111	17.5	39.6	49.5	75.3	0.79	1.22	0.64 (0.42–0.99)	0.042*
	Rectal findings	77/523	22/111	28.6	19.8	86.7	80.0	1.48	0.93	1.60 (0.93–2.77)	0.088
	Radiological findings	96/523	38/111	39.6	34.2	85.9	82.9	2.43	0.77	3.18 (1.97–5.14)	<0.001*
	Other alarm symptoms	188/523	39/111	20.7	35.1	63.8	78.5	0.97	1.02	0.96 (0.62–1.48)	0.841
	Positive FIT	239/283	45/48	18.8	93.8	17.4	93.2	1.14	0.36	3.17 (0.94–10.70)	0.051

CI, confidence interval; CRC, colorectal cancer; FIT, faecal immunochemical test; LR, likelihood ratio; NPV, negative predictive value; OR, odds ratio; PPV, positive predictive value; y, years; * = statistical significant difference.

In the adjusted logistic regression model without FIT, radiological findings and rectal findings showed the strongest adjusted associations with CRC. Age, male sex, visible blood and anaemia were also independently associated with CRC. Altered bowel habits were not associated with CRC in unadjusted analyses or in the main adjusted model. In the complete-case FIT model, altered bowel habits showed a weak adjusted association with CRC, but this finding should be interpreted cautiously because it was not supported by unadjusted diagnostic measures and was limited to patients with available FIT data.

In the complete-case model including FIT, positive FIT showed the strongest adjusted association with CRC. Radiological findings, rectal findings, anaemia, visible blood, altered bowel habits and age remained associated with CRC, whereas male sex and other alarm symptoms were not independently associated with CRC (Table 3).

**Table 3.** Multivariable logistic regression for CRC in the full cohort and in patients with available FIT data.

Predictor	aOR (95% CI)	*p-*Value
Main model, full cohort (*n* = 3258)		
Age, per year	1.03 (1.02–1.04)	<0.001*
Male sex	1.28 (1.05–1.55)	0.013*
Visible blood	1.59 (1.23–2.05)	<0.001*
Anaemia	2.04 (1.61–2.57)	<0.001*
Altered bowel habits	1.23 (1.00–1.52)	0.052
Rectal findings	3.58 (2.79–4.60)	<0.001*
Radiological findings	4.47 (3.44–5.82)	<0.001*
Other alarm symptoms	1.22 (0.99–1.51)	0.061
Complete-case FIT model (*n* = 1740)		
Age, per year	1.01 (1.00–1.03)	0.031*
Male sex	1.02 (0.77–1.36)	0.883
Visible blood	1.72 (1.19–2.51)	0.004*
Anaemia	2.22 (1.60–3.08)	<0.001*
Altered bowel habits	1.48 (1.08–2.03)	0.014*
Rectal findings	2.56 (1.71–3.82)	<0.001*
Radiological findings	5.60 (3.53–8.89)	<0.001*
Other alarm symptoms	1.21 (0.88–1.64)	0.237
Positive FIT	10.13 (4.92–20.83)	<0.001*

The main model included all patients in the analytical cohort. The FIT model was restricted to patients with available FIT results.

aOR, adjusted odds ratio; CI, confidence interval; CRC, colorectal cancer; FIT, faecal immunochemical test; * = statistical significant difference.

## Discussion

In this regional SCC-CRC referral cohort, no CRC cases were observed in patients aged 18–40 years, and the youngest patient with CRC was 41 years. Haemorrhoids were the most common finding in the 18–40 age group, suggesting that rectal bleeding is not predictive of cancer in that age group. These findings also show that the pattern of referral factors differs between age groups, with visible blood and other alarm symptoms more common in younger patients and altered bowel habits more common in older patients. Because the young subgroup was small and CRC is rare in this age range, the absence of CRC cannot be interpreted as proof that SCC-CRC referral is unnecessary in younger adults.

Data from The National Board of Health and Welfare show that the incidence of CRC in people aged 20–39 ranged from 3.82 people per 100,000 to 4.85 per 100,000 between 2016 and 2023 in Sweden.^15^ These data represent population incidence, whereas the data from our study reflect the prevalence of CRC in a symptomatic SCC-CRC referral cohort; therefore, the two datasets cannot be compared directly.

In contrast, our SCC-CRC referral cohort revealed that CRC was detected in 6.5% of patients aged 41–50 years and 11.2% of patients aged 51–60 years, demonstrating that clinically relevant CRC is present in symptomatic patients below the current screening age of 60 years. However, our cohort reflects a selected symptomatic referral cohort and cannot directly be translated into population screening age thresholds. These proportions are consistent with national Swedish data, where the incidence of CRC was 22 per 100,000 in individuals aged 40–49 years compared with 56 per 100,000 in those aged 50–59 years during 2016–2023.^15^ The prevalence of HRA and HGD polyps was similar in the 41–50 and 51–60 years age groups; these findings align with previous studies reporting no significant difference in adenoma detection rates between adults aged 45–49 and 50–54 years.^3^ Visible blood was not significant for any pathological finding, except for a negative association with HGD polyps in patients aged 71–80 years, indicating that visible blood is a weak and nonspecific predictor of clinically relevant colorectal pathology.

Several referral criteria associated with CRC were also associated with pathological findings in general, suggesting limited specificity for CRC. This overlap suggests that these criteria reflect colorectal pathology rather than malignancy specifically. After adjustment for age, sex and coexisting referral factors, radiological findings, rectal findings, anaemia and visible blood remained associated with CRC. However, sensitivity was low for most individual criteria, and PPV increased with age partly because CRC prevalence increased with age. Other alarm symptoms in the 61–70 age group had a low predictive value and a negative association with HRA, and no other significant associations were observed, suggesting that these symptoms are unreliable predictors of disease. Although other alarm symptoms are not included in the SCC criteria, they are commonly mentioned and can be a reason for starting a SCC-CRC referral; therefore, it is important to evaluate their value for predicting CRC and other pathologies in the colorectal tract.

Previous studies report that anaemia, radiological findings and positive FIT have a PPV for CRC.^16–18^ Our results are mostly consistent with these findings; however, anaemia was only significant in patients aged >80 years, where radiological findings also showed the highest PPV for CRC in any age group, suggesting greater specificity for cancer in older individuals. FIT was not significantly associated with CRC in patients over 80 years; a probable explanation is limited FIT availability. Radiological findings were coded only as present or absent because the dataset did not specify imaging method, finding type or level of diagnostic suspicion. This limits interpretation because an obvious colonic mass and nonspecific wall thickening are clinically different findings. FIT was available in only about half of patients, and patients with and without FIT differed in CRC prevalence and referral-factor distribution.

In many organised screening programmes, invitations begin at 50 years (often up to 74 years), in line with EU recommendations that specify FIT for people aged 50–74.^19,20^ Outside Europe, the United States Preventive Services Task Force recommends average-risk screening from 45 years.^21,22^ In the Nordic region, Denmark invites 50–74 every 2 years, whereas Finland launched nationwide screening in 2022 and currently targets 60–70, with planned expansion to 56–74. Sweden’s national FIT programme is being rolled out for 60–74 years (biennial), with full build-out planned by 2026.^7,23,24^ Our data show a significant CRC burden among selected symptomatic patients aged 41–60 years, but do not provide evidence for changing screening age. Screening decisions require population-based incidence data, expected benefits and harms, colonoscopy capacity and health-economic analyses.

A major strength of this study is the large, real-world SCC-CRC referral cohort, which reflects routine clinical practice within a standardised diagnostic pathway. Detailed age-stratified analyses provide clinically relevant information on how cancer yield and other pathological findings differ across age groups. Outcomes were based on colonoscopy findings, which are directly relevant to patient care. A limitation of this study is the small sample size in the youngest age group, which limited power for age-specific analyses. In addition, FIT data were not available for around half of the patients, and FIT testing was not randomly distributed, because patients without FIT data had a higher CRC frequency and more frequent radiological and rectal findings. Therefore, FIT-related diagnostic accuracy estimates and the complete-case FIT regression model should be interpreted cautiously. Other limitations include the absence of detailed radiology data, possible coding errors in routine clinical data and a lack of external validation. The regression models assess associations and should not be used for individual risk estimation without further validation.

## Conclusion

In this regional SCC-CRC referral cohort, no CRC was detected in patients aged 18–40 years. The frequency of referral factors varied across age groups, whereas the PPV for CRC increased with age. Several SCC-CRC referral criteria were associated with both CRC and other pathological findings, which indicates limited specificity for cancer. Several referral factors were independently associated with CRC, especially radiological findings, rectal findings, anaemia and FIT positivity. FIT showed the highest predictive value in our cohort, and published symptomatic-cohort evidence shows very high NPV for CRC at common thresholds; therefore, routine FIT testing in SCC-CRC referrals and complete FIT data capture should be evaluated to improve risk stratification and prioritisation of colonoscopy investigation.

## Supplemental Material

sj-docx-1-tag-10.1177_17562848261460138 – Supplemental material for Age-stratified colonoscopy outcomes and referral-factor associations in the Swedish colorectal cancer fast-track pathway: a retrospective cohort study Supplemental material, sj-docx-1-tag-10.1177_17562848261460138 for Age-stratified colonoscopy outcomes and referral-factor associations in the Swedish colorectal cancer fast-track pathway: a retrospective cohort study by Abdullah Jajan and Michiel van Nieuwenhoven in Therapeutic Advances in Gastroenterology

## Appendix

### Abbreviations

aOR adjusted odds ratio

CI confidence interval

CRC colorectal cancer

FIT faecal immunochemical test

HGD high-grade dysplasia

HRA high risk adenoma

LR likelihood ratio

NPV negative predictive value

OR odds ratio

PPV positive predictive value

RÖC Region Örebro County

SCC standardised course of care

SD standard deviation

## Declarations

**Ethics approval and consent to participate:** The study was approved by the Swedish Ethical Review Authority (Dnr. 2023-03776-02). The requirement for informed consent was waived by the Swedish Ethical Review Authority because of the retrospective design, minimal risk and use of de-identified data before analysis.

**Consent for publication:** Not applicable. The manuscript does not contain identifiable individual participant data.

**Availability of data and materials:** The data are not publicly available because they contain information that could compromise participant privacy and are subject to Swedish data protection regulations. De-identified data may be available from the corresponding author on reasonable request and with relevant approvals.*
