# Supplementary material for: Age-stratified colonoscopy outcomes and referral-factor associations in the Swedish colorectal cancer fast-track pathway: a retrospective cohort study
Source: Ther Adv Gastroenterol. 2026 Jun 28;19:17562848261460138. doi: 10.1177/17562848261460138 (PMC13311249; doi:10.1177/17562848261460138)
Supplement: sj-docx-1-tag-10.1177_17562848261460138 – Supplemental material for Age-stratified colonoscopy outcomes and referral-factor associations in the Swedish colorectal cancer fast-track pathway: a retrospective cohort study [file sj-docx-1-tag-10.1177_17562848261460138.docx]

# STROBE checklist for cohort studies

Manuscript: Age-stratified colonoscopy outcomes and referral-factor associations in the Swedish colorectal cancer fast-track pathway: a retrospective cohort study

| **Item** | **Recommendation** | **Reported in manuscript** |
| --- | --- | --- |
| Title and abstract | Indicate study design and provide informative abstract | Title and Abstract |
| Background/rationale | Explain scientific background and rationale | Introduction |
| Objectives | State specific objectives | End of Introduction |
| Study design | Present key elements of study design | Methods, Study design and reporting |
| Setting | Describe setting, locations and dates | Methods, Setting and patient selection |
| Participants | Describe eligibility criteria and selection | Methods, Setting and patient selection; Figure 1 |
| Variables | Define outcomes, exposures and covariates | Methods, Variables and definitions |
| Data sources/measurement | Give sources of data and assessment methods | Methods, Variables and definitions |
| Bias | Describe efforts to address bias | Methods, Missing data; Discussion |
| Study size | Explain study size | Methods, Statistical analysis |
| Quantitative variables | Explain handling of quantitative variables | Methods, Statistical analysis |
| Statistical methods | Describe statistical methods | Methods, Statistical analysis |
| Participants | Report numbers at each stage | Results, Figure 1 |
| Descriptive data | Give characteristics of participants | Results, Table 1 |
| Outcome data | Report outcome events | Results, Table 1 |
| Main results | Give unadjusted and adjusted estimates | Results, Tables 2-3 |
| Other analyses | Report additional analyses | Results, FIT complete-case model and missing-data comparison |
| Key results | Summarise key results with objectives | Discussion |
| Limitations | Discuss limitations | Discussion |
| Interpretation | Give cautious interpretation | Discussion and Conclusions |
| Generalisability | Discuss external relevance | Discussion |
| Funding | Give source of funding | Declarations |
